# Internalized-stigma and dissociative experiences in bipolar disorder

**DOI:** 10.3389/fpsyt.2022.953621

**Published:** 2022-07-29

**Authors:** Renato de Filippis, Giulia Menculini, Martina D'Angelo, Elvira Anna Carbone, Alfonso Tortorella, Pasquale De Fazio, Luca Steardo

**Affiliations:** ^1^Psychiatry Unit, Department of Health Sciences, University Magna Graecia of Catanzaro, Catanzaro, Italy; ^2^Department of Psychiatry, University of Perugia, Perugia, Italy; ^3^Psychiatry Unit, Department of Medical and Surgical Sciences, University Magna Graecia of Catanzaro, Catanzaro, Italy

**Keywords:** bipolar disorder, dissociation, internalized stigma, mental health, mood disorders, psychopathology, quality of life, trauma

## Abstract

**Introduction:**

Dissociative symptoms have been recently related to bipolar disorder (BD) symptomatology. Moreover, the disease burden carries on a share of perceived self-stigma that amplifies the BD impairment. Internalized stigma and dissociative symptoms often seem overlapping, leading toward common outcomes, with reduced treatment seeking and poor adherence. We hypothesize a potential relationship between dissociation and self-stigma in patients suffering from BD.

**Materials and methods:**

In this cross-sectional study we enrolled a total of 120 adult clinically stable BD outpatients. All participants completed the Internalized Stigma of Mental Illness (ISMI), Dissociative Experiences Scale-II (DES-II), and Manchester Short Assessment of Quality of Life (MANSA).

**Results:**

Average age and age at BD (BD-I *n* = 66, 55%; BD-II *n* = 54, 45%) onset were 46.14 (±4.23), and 27.45 (±10.35) years, with mean disease duration of 18.56 (±13.08) years. Most participants were female (*n* = 71; 59.2%) and 40 (33%) of them experienced lifetime abuse, with an average of 1.05 (±0.78) suicide attempts. DES scores (mean 31.8, ±21.6) correlated with ISMI total-score, with significant association with spikes in Alienation (13.1, SD±3.1) (*p* < 0.001) and Stereotype (13.8, SD±3.9) (*p* < 0.001). Linear regression analysis has shown a significant association between DES total score and alienation (*p* < 0.001), stereotype (*p* < 0.001) and MANSA total-score (*p* < 0.001).

**Discussion:**

For the first time, our data suggests that self-stigma is associated to dissociative symptoms, reducing overall quality of life in BD. The early identification of at-risk patients with previous lifetime abuse and high perceived stigma could lead the way for an ever more precise tailoring of treatment management.

## Introduction

Bipolar disorder (BD) is a severe psychiatric disorder characterized by shifts of emotions, energy and thought, as well as changes in concentration and sleep need, with impairment in the ability to carry out day-to-day tasks, which mainly occur during biphasic mood episodes of mania or hypomania and depression, and are expressed as recurrent episodes of changes in energy levels and behavior, with a negative impact on patients' overall quality of life (1, 2).

The lifetime prevalence is around 1% worldwide, and, usually, the onset is in the late second or early third decade of life (1, 3). The chronic episodic course negatively affects several aspects of patients' life, including interpersonal relationships and occupational functioning, and can also lead to severe outcomes, including death by suicide (4, 5). Recently, dissociative symptoms have been related to BD symptomatology and phenotype characterization suggesting a role in the etiology and clinical course of the disease, thus representing a new area of study both for research and for the clinic, with the aim of a more precise tailoring of patients' treatment management (6).

Frequently, not recognizing the mood shift and poor insight can delay treatment initiation and a worsening of the clinical course (7, 8). Indeed, these two aspects of BD could be due to a considerable heterogeneity of clinical presentation and the patients' fear of being stigmatized in case of seeking help (9). However, both reduced treatment-seeking and the worst clinical course can lead to a severe outcome and frequently are associated with social withdrawal, functional impairment and alienation (4, 5). Consequentially, stigma is a critical issue in BD because it leads to poor treatment adherence and more severe symptomatology (10). This is an important concern because the chronic mood shift could represent a risk factor for psychosis, traumatic events, and cognitive impairment, and all those aspects were correlated to dissociative symptomatology (11, 12).

Moreover, several studies highlight that patients suffering from affective disorders present dissociation as a preferred coping strategy (13, 14), and dissociative experience disrupts wholeness in the stream of mind. Dissociation also prevents the integration of experiences and information and leads to amnesia, depersonalization, and derealization (15, 16). Additionally, internalized stigma and dissociative symptoms seem to overlap, sharing features such as alienation, isolation, functional impairment and disease burden, often leading to common outcomes with reduced treatment-seeking and poor adherence (17).

In recent years, some advances have been made regarding the knowledge concerning stigma in BD, and, according to our current understanding, there are mainly three kinds of stigma. The most well-studied concept is the internalized or self-stigma, which explains the subjective appreciation of negative experiences and perceptions of the patients themselves, leading to identity transformation and stereotype endorsement (18). The second entity is perceived stigma, namely the patients' subjective experience of being stigmatized by other agencies (19, 20). This is mostly contributed by endorsing various discriminatory traits deep-rooted in the disease process. Structural or systemic stigma is the third and probably a minor studied entity. It refers to institutional policies and practices surrounding a person that creates inequality by restricting opportunities for people suffering from mental illness (21, 22). Stigma also involves perceiving patients with BD with a negative outlook and attributing stereotypes, thus further leading to interference in community participation. However, the debate on developing effective interventions to fight stigma related to BD or other general medical and psychiatric diseases is still ongoing (23).

We hypothesize a potential relationship between internalized stigma and dissociative phenomena in patients affected by BD, potentially leading to a peculiar negative clinical course and a worsening quality of life. Therefore, the present work aimed to assess the correlation between self-stigma, dissociative symptoms, and quality of life in a clinical sample of patients suffering from BD with a cross-sectional study design, and to test if the dissociative symptomatology may be related to a specific subthreshold of internalized stigma and/or to quality of life.

## Materials and methods

### Participants and procedures

This study was designed as a naturalistic and uncontrolled cross-sectional observational study at the outpatients' Psychiatry unit of the University Hospital Mater Domini of Catanzaro (Italy) and was conducted between May 2020 and January 2022. All consecutive potentially candidate patients were screened for eligibility and invited to participate in the study, where applicable. Participants were screened and diagnosed by a clinical interview conducted by experienced clinicians through the Structured Clinical Interview for DSM-5 (SCID-5 CV) (24). The interviewers were experienced psychiatrists who work in clinical research, were trained in administering neuropsychiatric tools, and used these tests in their daily clinical practice.

We included all patients fulfilling the following inclusion criteria: (1) aged between 18 and 70 years and able to read and understand the informed consent form; (2) capability to answer self-report questionnaires; (3) diagnosed with BD type-I (BD-I) or type-II (BD-II) according to the Diagnostic and Statistical Manual of Mental Disorders-fifth edition (DSM-5) (24); (4) clinically stable, if at the time of enrollment the Clinical Global Impression for Bipolar Patients (CGI-BP) (25) scored ≤2 at item 1 (severity of illness). We did not set any other inclusion criteria, with the goal to achieve a real-world clinical sample, as routinely visited in daily clinical activity. Regarding exclusion criteria, we excluded patients if: (1) with recent (≤6 months) or uncertain BD diagnosis or with a medical history that was implausible or undocumented; (2) with comorbid psychiatric diagnosis (i.e., schizophrenia spectrum disorders, major depressive disorder, post-traumatic stress disorder); (3) affected by dementia or intellectual disability from mild to severe according to DSM-5 (corresponding to IQ <70); (4) with alcohol or drug abuse in the previous 6 months and dependence for 12 months according to DSM-5 diagnostic criteria; (5) suffering from another severe medical condition related with psychiatric symptoms (e.g., temporal lobe epilepsy, multiple sclerosis, brain trauma, malignant disease).

According to the Ethical Committee, participants were provided with a complete description of the study aims and methods and gave written informed consent to participate in the study before any procedure took place. The study protocol was submitted and approved by the Ethical Committee of University Hospital Mater Domini at Catanzaro (n. 307/2020), and the study procedures were carried out in accordance with the ethical principles set out in the revised version of the Helsinki Declaration (26).

### Assessments

We collected patients' demographics and clinical (i.e., psychopharmacological therapy) information through an *ad hoc* schedule. In detail, we used a semi-structured interview collecting data on age, sex, civil status, years of education, current occupation, family history of psychiatric diseases, psychiatric and general medical comorbidity, onset, and longitudinal course of the disorder (e.g., number of depressive/hypo/manic episodes, mixed and anxious features, and psychotic symptoms), number of previous suicidal attempts and psychiatric hospitalizations, and current prescribed treatments.

Then, all participants were evaluated by means of the following Italian versions of assessment scales:

• The Clinical Global Impression for Bipolar Patients (CGI-BP) (25) is a modified version of the original CGI specifically developed to assess global illness severity and change in patients affected by bipolar disorder, and already used with Italian clinical samples (27). It is divided into two sections, severity of illness and global improvement, and both range between a minimum of one (“normal, not ill at all”) to a maximum of seven (“among the most extremely ill patients”), while 0 denotes the impossibility to assess the score.

• The Internalized Stigma of Mental Illness (ISMI) scale (28) is a 29-item self-administrated questionnaire measuring self-stigma in the population suffering from mental disorders. It has been structured to quantity the subjective experience of stigma, with subscales measuring Alienation (six items), Stereotype Endorsement (seven items), Perceived Discrimination (five items), Social Withdrawal (six items) and Stigma Resistance (five items) through 29 Likert questions with four reply options, ranging between strongly disagree (one point) and strongly agree (four points), with a total score between 29 and 116 (28). It is a widely used and validated tool whose psychometric proprieties have been comprehensively evaluated across multiple versions, cultures, and languages, including Italy (18, 29), as well as several major psychiatric disorders (e.g., depression, schizophrenia, substance abuse, eating disorders) and general medical illnesses (e.g., epilepsy, inflammatory bowel disease, leprosy) (20). It should be considered that the five stigma resistance subscale items are reverse-coded, and also serve as a validity check (28). Therefore, stigma resistance displays the same direction of correlation as the other four subscales. A high total score on the ISMI scale indicates more severe internalized stigmatization (30).

• The Dissociative Experiences Scale II (DES-II) (31), and its Italian version (32), is a largely used self-assessment measure developed to offer a feasible tool of reliably quantifying dissociative symptoms in both general and clinical populations. The scale is made up of 28 items describing dissociation features (i.e., absorption, amnesia, depersonalization, and derealization), and the user is asked to select a percentage defining how much the patient experienced the symptom, ranging from 0 (never) to 100% (always). The final score comes from the sum of all items divided by the number of total items (i.e., 28), ranging between 0 and 100. The dissociative disorder cut-off is settled with scores >30.

• The Manchester Short Assessment of Quality of Life (MANSA) has been developed as a brief, handy and innovative instrument for assessing the quality of life, focusing on satisfaction with life as a whole and with life domains (33). It includes 16 questions, four of them investigating the objective quality of life and rated by a dichotomized yes/no scale, and 12 rated on a 7-point scale and exploring satisfaction with life, job, financial situation, friendships, leisure activities, accommodation, personal safety, people that the person lives with, family and global health. Its maximum total score is 93 points. Finally, an overall subjective quality of life score may be calculated (34). The MANSA scale showed good reliability, construct validity, and internal consistency when investigating quality of the life in people affected by severe general medical and psychiatric conditions, including Italian samples (34, 35).

### Statistical analyses

Descriptive statistics were calculated for socio-demographic and clinical characteristics and other relevant assessment instruments. As appropriate, data are presented as means and standard deviations (SD) or frequencies and percentages (%). A Spearman's correlation analysis was performed to test the correlation between psychometric scale and clinical and sociodemographic variables. A linear regression model was used to describes the relationship between the DES total score as dependent variable, and ISMI subscales and MANSA total score as independent variables. Odds ratios (OR) with 95% confidence intervals were assessed for observed associations. All tolerance values in the regression analyses were >0.1 and all variance inflation factors were <10, expressing that the assumption of multicollinearity was not violated. The level of statistical significance was set at a nominal value of *p* ≤ 0.05. Statistical analyses were performed by using the Statistical Package for Social Sciences Version 26 (SPSS, Chicago, Illinois, USA).

## Results

We approached a total of 138 consecutive patients fulfilling the inclusion and exclusion criteria. Of these, a total of 18 did not participate in the study due to the following reasons: refusal to complete the assessment (*n* = 10) or to sign the informed consent (*n* = 6), or other reasons (*n* = 2). Therefore, the final sample was made up of 120 patients, of whom 66 (55%) suffering from BD-I and 54 (45%) from BD-II. The average age (± standard deviation, SD) was 46.1 (±14.23) years, and the majority of participants were female (*n* = 71; 59.2%), single (*n* = 56; 46.7%), graduated (*n* = 93; 73.5%), employed (*n* = 70; 58.3%), with positive family history for both psychiatric (*n* = 82; 68.3%) and general medical (*n* = 69; 57.5%) disease, and 33.3% (*n* = 40) of them experienced lifetime abuse (Table 1).

**Table 1:** Demographics and personal characteristics of the sample.

		***N*** = **120**	
Age^a^		46.14	(14.23)
Gender^b^	Men	49	(40.8)
	Women	71	(59.2)
Education (years)^a^		13.45	(3.37)
Graduated^b^	Yes	93	(73.5)
Civil status^b^	Single	56	(46.7)
	Married	44	(36.7)
	Divorced	16	(13.3)
	Widow	4	(2.5)
Occupation^b^	Employed	70	(58.3)
	Unemployed	33	(27.5)
	Students	17	(14.2)
Diagnosis^b^	Bipolar disorder type I	66	(55.0)
	Bipolar disorder type II	54	(45.0)
Family history of psychiatric disorders^b^	Positive	82	(68.3)
Family history of general medical diseases^b^	Positive	69	(57.5)
Lifetime abuse^b^	Yes	40	(33.3)

a * Data are presented as means (SD)*.

b *Data are presented as frequencies (%)*.

Regarding clinical features, the average age (± standard deviation, SD) at BD onset was 27.45 (±10.35), while the average disease duration was 18.56 (±13.08) years. We recorded a mean of 5.43 (±5.10) depressive, 3.92 (±2.90) maniac, and 3.11 (±3.06) hypomanic episodes, then the average number of affective episodes was 10.47 (±9.91) among patients. Most included patients presented aggressive behaviors (*n* = 70; 58.3%) and anxious features (*n* = 79; 65.8%), with also frequent mixed (*n* = 59; 49.2%) and psychotic (*n* = 53; 44.2%) symptoms. Seasonality was also very common (*n* = 56; 46.7%), as well as suicidality (*n* = 39; 32.5%), with an average of 1.05 (±0.78) suicide attempts (Table 2).

**Table 2:** Clinical features of the sample.

	***N*** = **120**	
Age of BD onset (years)^a^	27.45	(10.35)
Age first psychiatric contact (years)^a^	30.16	(10.57)
Age of first admission (years)^a^	29.86	(9.4)
Age of first hypomanic episode (years)^a^	31.47	(10.34)
Age of first depressive episode (years)^a^	27.76	(10.43)
Age of first manic episode (years)^a^	28.50	(7.70)
Duration of the disease (years)^a^	18.56	(13.08)
Duration of hospitalization (days)^a^	9.93	(5.93)
Number of depressive episodes^a^	5.43	(5.10)
Number of manic episodes^a^	3.92	(2.90)
Number of hypomanic episodes^a^	3.11	(3.06)
Lifetime number of affective episodes^a^	10.47	(9.91)
Number of affective episodes in the last year^a^	0.78	(0.78)
Number of suicide attempts^a^	1.05	(0.78)
Seasonality^b^	56	46.7
Suicidality (positive)^b^	39	32.5
Aggressive behaviors (positive)^b^	70	58.3
Mixed features (positive)^b^	59	49.2
Anxious features (positive)^b^	79	65.8
Psychotic symptoms (positive)^b^	53	44.2

a * Data are presented as means (SD)*.

b *Data are presented as frequencies (%)*.

The DES mean (± standard deviation, SD) score was higher than the settled cut-off of 30 points (i.e., 31.8 (±21.6), with spikes shown especially in Alienation (13.1, ±3.1), Stereotype (13.8, ±3.9) and Social distancing (13.0, ±2.7) items. As for the MANSA scale, participants scored an average total of 49.1 (±10.9) out of 93 total points, while ISMI mean score was 60.4 (±9.6) out of a tool range between 29 and 116 (Table 3).

**Table 3:** Assessment evaluation.

	***N*** = **120**	
DES total score	31.75	(21.61)
ISMI total score	60.40	(9.62)
Alienation	13.07	(3.06)
Stereotype	13.83	(3.92)
Discrimination	9.86	(2.17)
Social withdrawal	12.99	(2.74)
Stigma resistance	8.21	(1.25)
MANSA total score	49.14	(10.94)

*All values are reports as mean (± standard deviation)*.

Table 4 includes the results of Spearman's correlations between DES-II total score, ISMI-specific subdomains, ISMI total score and MANSA total score and clinical features (i.e., number of total episodes, hospitalization, psychotic features, and previous substance abuse). Several significant correlations emerged for almost all variables, particularly between DES total score, ISMI subdomains and ISMI total score.

**Table 4:** Results of Spearman correlation analysis.

	**DES total score**	**Alienation**	**Stereotype**	**Discrimination**	**Social withdrawal**	**Stigma resistance**	**ISMI total score**	**MANSA total score**	**n. of total episodes**	**Hospitalization (*n*)**	**Psychotic features**	**Previous substance abuse**
DES total score	–	^**−**^	^**−**^	^**−**^	^**−**^	^**−**^	^**−**^	^**−**^	^**−**^	^**−**^	^**−**^	^**−**^
Alienation	0.865^**^	–	^**−**^	^**−**^	^**−**^	^**−**^	^**−**^	^**−**^	^**−**^	^**−**^	^**−**^	^**−**^
Stereotype	0.901^**^	0.844^**^	–	^**−**^	^**−**^	^**−**^	^**−**^	^**−**^	^**−**^	^**−**^	^**−**^	^**−**^
Discrimination	0.632^**^	0.615^**^	0.687^**^	–	^**−**^	^**−**^	^**−**^	^**−**^	^**−**^	^**−**^	^**−**^	^**−**^
Social withdrawal	0.626^**^	0.675^**^	0.649^**^	0.671^**^	–	^**−**^	^**−**^	^**−**^	^**−**^	^**−**^	^**−**^	^**−**^
Stigma resistance	−0.183*	**–**0.101	−0.175	−0.199*	**–**0.103	–	^**−**^	^**−**^	^**−**^	^**−**^	^**−**^	^**−**^
ISMI total score	0.811^**^	0.895^**^	0.879^**^	0.778^**^	0.834^**^	−0.012	–	^**−**^	^**−**^	^**−**^	^**−**^	^**−**^
MANSA total score	−0.851^**^	−0.743^**^	−0.801^**^	−0.531^**^	−0.573^**^	0.139	−0.718^**^	–	^**−**^	^**−**^	^**−**^	^**−**^
n. of total episodes	0.540^**^	0.482^**^	0.508^**^	0.325^**^	0.361^**^	−0.141	0.460^**^	−0.403^**^	–	–	–	–
Hospitalization (*n*.)	0.616^**^	0.515^**^	0.514^**^	0.486^**^	0.537^**^	−0.188^*^	0.539^**^	−0.570^**^	0.497^**^	–	–	–
Psychotic features	0.774^**^	0.712^**^	0.719^**^	0.576^**^	0.662^**^	−0.246^**^	0.701^**^	−0.741^**^	0.530^**^	0.784^**^	–	–
Previous substance abuse	0.416^**^	0.452^**^	0.401^**^	0.243^**^	0.336^**^	−0.035	0.379^**^	−0.419^**^	0.251^**^	0.543^**^	0.419^**^	–

* *p < 0.05*;

** *p < 0.01. Significant results are in bold*.

A linear regression with DES total score as dependent variable was performed to assess the association between dissociative symptomatology and ISMI total score, ISMI sub-domains, and MANSA. A significant association was found between Alienation (B = 0.279; *t* = 4.329; *p* < 0.001), Stereotype (B = 0.331; *t* = 4.555; *p* < 0.001) and MANSA total score (B = **–**0.320; *t* = **–**5.909; *p* < 0.001) (Table 5).

**Table 5:** Results of linear regression.

**Independent variable**	**Dependent variable**	**Not Standardized coefficients**		**Standardized coefficients**	
		**B**	**Standard error**	**B**	***t***	***p***
Alienation	DES total score	1.969	0.455	0.279	4.329	**<0.001**
Stereotype endorsement	DES total score	1.822	0.400	0.331	4.555	**<0.001**
Perceived discrimination	DES total score	0.756	0.474	0.076	1.595	0.113
Social withdrawal	DES total score	0.119	0.391	0.015	0.306	0.760
Stigma resistance	DES total score	−1.849	0.565	−0.107	−3.274	**<0.001**
MANSA total score	DES total score	**–**0.633	0.107	−0.320	−5.909	**<0.001**

*Significant results are in bold*.

## Discussion

This study found a strong relationship between internalized stigma and dissociative phenomena in patients suffering from BD, which may lead to a peculiar negative clinical course and a worsening quality of life. To the best of our knowledge, this is the first time internalized stigma was explored concerning its role in dissociation symptoms in BD, with consequences on quality of life. Further, as shown by Spearman's correlation analysis, internalized stigma is correlated to several clinical variables predictive of poor clinical outcomes in BD. ISMI total score correlates to a higher number of total episodes, hospitalization, psychotic features, and previous substance abuse, highlighting its impact on those factors predictive of a higher psychopathological burden. Such results are easily explained due to the impact of stigma on the life of psychiatric patients, especially those suffering from BD (36, 37). A growing body of literature focused on the sociodemographic and clinical variables correlated to the stigma, and our results are in line with them except for the age of onset, which was poorly investigated before (38, 39). Stigma has a significant impact on people with BD, linked to negative stereotypes, prejudice and discrimination (8).

Usually, one of the most explored findings resulting from studies using ISMI is the relationship between internalized stigma and more severe psychopathology, lower self-esteem, reduced treatment adherence, and greater symptom severity (40–42). Pilot studies exploring ways to reduce internalized stigma are promising and warrant further investigation (20). In addition, a negative correlation is reported with the perceived quality of life. Initial studies on ways to reduce internalized stigma are promising but need further investigation (43, 44). Research has focused on the relationship between internalized stigma and self-esteem and how this implies negative evaluations (45, 46). An issue underlying this hypothesis is the influence that different processes have on self-esteem: one of the most critical concerns is the response of others. Many authors suggest that patients affected by mental illness tend to have dissociative symptoms that lead to alienation, discrimination, and negative representation even before being diagnosed. Due to this, several negative consequences occur, including the tendency to social isolation and a reduction in interpersonal relationships. This maladaptive schema worsens the patient's quality of life (47, 48). To date, little research has focused on the phenomenon of internalized stigma and the negative aspects related to it. The results obtained in the present study confirm our hypothesis, according to which there is a direct effect between dissociation and internalized stigma, particularly in the alienation and stereotype fields. Alienation is widespread among patients with bipolar disorder, especially those with higher psychopathological burdens, and refers to a sense of self-estrangement and poor social connection (49). The phenomenon of alienation represents an essential indicator of mental well-being and is often associated with depressed mood, dissociative symptoms, and psychological distress, even in other severe psychiatric disorders (50). According to our results, patients with a high level of dissociative symptoms and low self-esteem attribute a high level of stigma to themselves.

Further, previous research indicates alienation as a maladaptive coping strategy in psychiatric patients facing traumatic experiences (51, 52). This field might explain the strong connection resulting in the linear regression between alienation and dissociation. Moreover, a significant association was found between dissociation and stereotype. These results support the hypothesis that mental illness stereotypes still may represent a cultural barrier. Indeed, patients affected by chronic severe psychiatric disorders could experience social stigma feeling it as a trauma, thus rising maladaptive coping strategies, including dissociation (53).

Consequently, both self-stigma and dissociative symptoms represent two elements of greater severity of BD that potentially worsen its clinical outcome lowering treatment adherence and deteriorating the prognosis (54, 55). Previous research has focused on the difference in internalized stigma in different cohorts of psychiatric patients, highlighting the major level of internalized stigma in bipolar disorder rather than non-affective psychotic disorders (56, 57). Another expected result in line with the literature is the negative relationship between internalized stigma and quality of life. Several studies have been conducted on this topic. The explanation is that internalized stigma is associated with adverse psychological outcomes such as depressive symptoms, lower self-esteem and reduced self-efficacy, which a poorer outcome will reflect on the psychological domain (58–60).

Moreover, results presented in our study should be interpreted in the light of both some limitations and strengths. Indeed, although the clinical sample involved was recruited in a naturalistic setting, adequate to describe the general population and similar to several analogous studies (8, 61, 62), the inconspicuous final sample size as well as the lack of hypothesis-driven sample size estimation and the absence of confounders addressed in the linear regression model represent the main study limitations which preclude drawing causal conclusions; therefore, we foresee the implementation of the sample size together with a prospective study design to confirm the results obtained. Secondly, the cross-sectional design using self-administered evaluations represents a structural limitation regarding the assembly and reliability of the data, which must be considered in any generalization of the results. Finally, the enrolled patients were clinically stabilized as inclusion criteria, so a mirror evaluation in patients with acute BD may result differently. Hence, the need to replicate similar protocols on larger samples with perspectives capable of acquiring designs at different stages of the disease. On the other hand, this was the first attempt to evaluate the role and implications of internal stigma in BD, taking into account its relationships to demographics, history of the disease, clinical features, quality of life, and dissociative symptoms. Thus, the results presented in this study open a new perspective on the role that self-stigma and dissociative symptoms play in BD. Indeed, future studies could shed the light on the causal and temporal relationship existing between internalized-stigma and dissociation, opening new and interesting frontiers in both clinical and research fields.

## Conclusion

Recently, dissociative symptoms have been studied concerning their impact on BD clinical course and treatment response, while perceived stigma is already well-known to interfere with clinical outcomes. However, the relationship between dissociative symptoms, self-stigma and quality of life in patients suffering from BD is still far from being fully understood and has been explored in this study.

Although burdened by several limitations and by a cross-sectional study design which avoids a generalization, our findings correlate self-stigma reported by patients affected by BD to experienced dissociative symptoms, resulting in a reduced overall quality of life. Therefore, the study of this network may represent an area of clinical research interest for the future, with the goal of reaching a more patients' focused clinical practice to anticipate a precise diagnosis, manage personalized treatment, and improve prognosis.

## Data availability statement

The raw data supporting the conclusions of this article will be made available by the authors, without undue reservation.

## Ethics statement

The studies involving human participants were reviewed and approved by Ethical Committee of University Hospital Mater Domini at Catanzaro (Italy) (n. 307/2020), and the study procedures were carried out in accordance to the ethical principles set out in the revised version of the Helsinki Declaration. The patients/participants provided their written informed consent to participate in this study.

## Author contributions

LS: conceptualization and formal analysis. RdF and LS: methodology and writing the original draft preparation. RdF, MD'A, and LS: investigation and data curation. RdF, GM, EC, AT, PD, and LS: writing and review and editing. All authors read and approved the final manuscript.

## Conflict of interest

The authors declare that the research was conducted in the absence of any commercial or financial relationships that could be construed as a potential conflict of interest.

## Publisher's note

All claims expressed in this article are solely those of the authors and do not necessarily represent those of their affiliated organizations, or those of the publisher, the editors and the reviewers. Any product that may be evaluated in this article, or claim that may be made by its manufacturer, is not guaranteed or endorsed by the publisher.
